# Interaction between the amount of dietary protein and the environmental temperature on the expression of browning markers in adipose tissue of rats

**DOI:** 10.1186/s12263-019-0642-x

**Published:** 2019-06-04

**Authors:** Gabriela Alemán, Ana Laura Castro, Ana Vigil-Martínez, Ivan Torre-Villalvazo, Andrea Díaz-Villaseñor, Lilia G. Noriega, Isabel Medina-Vera, Guillermo Ordáz, Nimbe Torres, Armando R. Tovar

**Affiliations:** 1 Department of Fisiología de la Nutrición, Instituto Nacional de Ciencias Médicas y Nutrición Salvador Zubirán, Ciudad de México, Av. Vasco de Quiroga No. 15, Col. Belisario Domínguez Sección XVI, 14080 México, D.F, Mexico; 2 Instituto de Investigaciones Biomédicas, UNAM, 04510 Mexico City, Mexico; 3 Department of Research Methodology, Instituto Nacional de Pediatría, 04530 Mexico City, Mexico

**Keywords:** Dietary protein, Browning, Cold exposure, FGF21, Brown adipose tissue

## Abstract

**Background:**

A low-protein diet increases the expression and circulating concentration of FGF21. FGF21 stimulates the browning process of WAT by enhancing the expression of UCP1 coupled with an increase in PGC1α. Interestingly, the consumption of a low-protein diet could stimulate WAT differentiation into beige/brite cells by increasing FGF21 expression and *Ucp1* mRNA abundance. However, whether the stimulus of a low-protein diet on WAT browning can synergistically interact with another browning stimulus, such as cold exposure, remains elusive.

**Results:**

In the present study, rats were fed 6% (low), 20% (adequate), or 50% (high) dietary protein for 10 days and subsequently exposed to 4 °C for 72 h. Body weight, food intake, and energy expenditure were measured, as well as WAT browning and BAT thermogenesis markers and FGF21 circulating levels. The results showed that during cold exposure, the consumption of a high-protein diet reduced UCP1, TBX1, *Cidea*, *Cd137*, and *Prdm16* in WAT when compared with the consumption of a low-protein diet. In contrast, at room temperature, a low-protein diet increased the expression of UCP1, *Cidea*, and *Prdm16* associated with an increase in FGF21 expression and circulating levels when compared with a consumption of a high-protein diet. Consequently, the consumption of a low-protein diet increased energy expenditure.

**Conclusions:**

These results indicate that in addition to the environmental temperature, WAT browning is nutritionally modulated by dietary protein, affecting whole-body energy expenditure.

**Graphical abstract:**

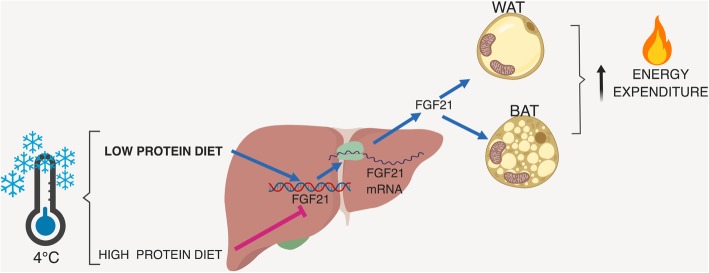

## Background

Adipose tissue in mammals is largely composed of two types of adipocytes, white adipocytes that form white adipose tissue (WAT) and brown adipocytes that form brown adipose tissue (BAT), and both tissues use glucose and fatty acids [1] to regulate energy storage and energy expenditure [2, 3]. Research in the last several years has shown that WAT can be induced to “browning” under circumstances, such as cold exposure, nutrient availability, or β-adrenergic stimulation [4–7]. The brown-like cells that appear in regular WAT have been called “beige” or “brite” (brown in white) adipocytes [8], characterized by an increase in uncoupling protein 1 (UCP1) expression along with specific markers, such as PRDM16 and CIDEA. Several pharmacological and nutritional stimuli have been related to the process of WAT browning, including PPARα or PPARγ agonists, methionine or leucine restriction, capsaicin, and high-fat diet [4, 9]; there is evidence that the amount of dietary protein can modify the browning process of WAT [10–12].

Recent studies have demonstrated that the amount of protein consumed in the diet modifies the expression and circulating concentration of fibroblast growth factor 21, termed FGF21. Evidence shows that the lower the amount of protein in the diet, the higher the expression of FGF21 in the liver [13, 14]. As a consequence, there is an increase in circulating FGF21 that stimulates hepatic gluconeogenesis and fatty acid oxidation, as well as an accelerated lipolysis in WAT [15]. In addition, the circulating concentration of FGF21 is increased by cold exposure [16–18]. Interestingly, adrenergic stimulation induces FGF21 expression in BAT instead of the liver [19]. Further evidence shows that FGF21 released from WAT auto-stimulates the browning of WAT by enhancing the expression of UCP1 coupled with an increase in PGC1α [16, 17]. However, recent evidence indicates that the beneficial effects of FGF21 are independent of the browning process [20].

Therefore, the present evidence indicates that the consumption of a low-protein diet can stimulate the transdifferentiation of WAT into beige/brite cells [11, 12], increase the expression of FGF21 [13, 21], and increase *Ucp1* mRNA abundance in WAT and BAT [12, 22]. Thus, it would be interesting to determine whether the potential stimulus of a low-protein diet on WAT browning can synergistically interact with other browning stimuli, such as cold exposure. Therefore, the aim of the present study was to assess whether a low- or high-protein diet could alter the expression of WAT browning markers and BAT thermogenesis, as well as the circulating levels and expression of FGF21 in the liver and BAT of rats at room temperature or exposed to a cold environment.

## Methods

### Animals

Male Wistar rats of 6 weeks of age were obtained from the Experimental Research Department and Animal Care Facility at the Instituto Nacional de Ciencias Médicas y Nutrición Salvador Zubirán and housed individually in stainless steel wire cages at 23 °C with a 12-h on/12-h off light-dark cycle (7:00 AM–7:00 PM) and free access to water. Institutional guidelines for animal care and use were followed. This protocol was approved by the Animal Care Committee of the Instituto Nacional de Ciencias Médicas y Nutrición Salvador Zubirán (CINVA-1792 FNU-1792-16/18-1).

### Dietary treatments

**Fig. 1:**
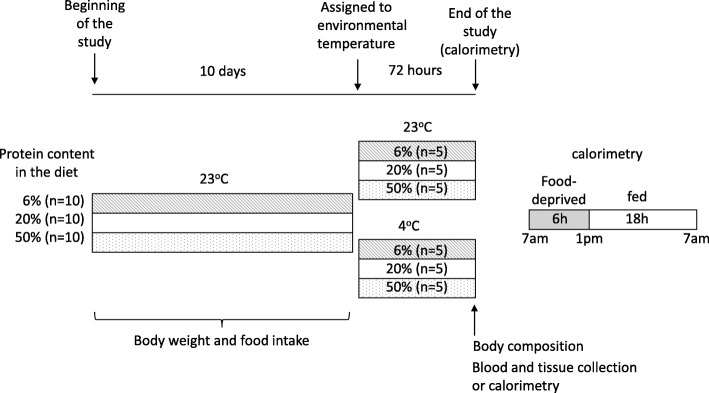
Experimental design timeline

Thirty rats of 6 weeks of age, and weighing 150–160 g, were divided into three subgroups of 10 rats, with each receiving one of the following isoenergetic experimental diets: (1) L: low-protein (6%); (2) A: adequate-protein (20%); or (3) H: high-protein (50%) on a restricted schedule (7 pm–7 am) to train the rats to consume their diets exclusively during this period. All rats were housed individually in stainless steel wire cages to quantify food intake. Diets were administered in dry form, and their composition was adjusted according to the recommendations of AIN-93 [23] (Table 1). After 10 days, rats from each group were randomly subdivided into two groups of 5 rats each: (1) the control group was maintained at room temperature (23 °C) as previously reported [24–28], and (2) the experimental group was maintained at a cold temperature (4 °C) for 72 h. Immediately after this period, body composition analysis was performed and the rats were killed. During the entire experiment, the rats had free access to water. The body weight and food intake were measured daily during the study period (Fig. 1).

**Table 1:** Composition of experimental diets (g/100 g)

Ingredient	Dietary protein concentration		
	6%	20%	50%
	g/%		
Casein (90.8% purity)^1^	6.608	22.026	55.066
L-cystine	0.18	0.18	0.18
tert-Butylhydroquinone	0.0014	0.0014	0.0014
Soy oil	7.0	7.0	7.0
Cornstarch	42.8	35.1	18.57
Dextrose	42.8	35.1	18.57
Vitamin mix^2^	0.1	0.1	0.1
Mineral mix^3^	0.5	0.5	0.5

^1^“Vitamin-free” casein, Harlan Teklad research diets. Casein amino acid concentration (g/100 g protein): Ala 2.8, Arg 3.4, Asp 6.3, Cys 0.3, Glu 20.5, Gly 1.6, His 2.5, Ile 4.7, Leu 8.2, Lys 7.2, Met 1.9, Phe 4.4, Pro 9.5, Ser 5.0, Thr 3.8, Trp 1.6, Tyr 4.7, and Val 6.0

^2^Teklad custom diet, AIN-93-VX. Formula (g/100 g): Niacin 0.3, calcium pantothenate 0.16, pyridoxine HCl 0.07, thiamin 0.06, riboflavin 0.06, folic acid 0.02, biotin 0.002, vitamin B_12_ (0.1% in mannitol) 0.25, vitamin E, DL-alpha tocopheryl acetate (500 IU/g) 1.5, vitamin A palmitate (500,000 IU/g) 0.08, vitamin D_3_, cholecalciferol (500,000 IU/g) 0.02, vitamin K_1_, phyloquinone 0.0075, sucrose, fine ground 97.47

^3^Rogers-Harper, Harlan Teklad research diets. Formula (g/100 g): potassium phosphate, monobasic 34.3, calcium carbonate 29.29, sodium chloride 25.06, magnesium sulfate, heptahydrate 9.98, ferric citrate 0.623, calcium phosphate, dibasic, dihydrate 0.43, ammonium paramolybdate, tetrahydrate 0.0025, cupric sulfate 0.156, manganese sulfate, monohydrate 0.121, potassium iodide 0.0005, sodium selenite 0.0015, zinc chloride 0.02

At the end of the study, the rats were introduced into a carbon dioxide chamber and euthanized by decapitation. The subcutaneous white fat pad (WAT) from the leg region, the interscapular BAT, and the liver were rapidly collected, frozen in liquid nitrogen, and stored at − 70 °C until further analysis. Samples of these tissues were also collected in formalin at room temperature for histological analysis. In addition, blood samples were collected and centrifuged at 1000×*g* for 10 min at 4 °C, and the serum was stored at − 70 °C until further analysis.

### Body composition and indirect calorimetry

The body composition was evaluated at the end of the study using magnetic resonance imaging (EchoMRI, Echo Medical Systems, Houston, TX, USA.) to measure lean and fat mass. The scannings were performed by introducing the animals in a thin-walled plastic cylinder (3 mm thick, 6.8 cm internal diameter), and a cylindrical plastic insert to limit the rat movement. While in the cylinder, the animals were briefly subjected to a low intensity electromagnetic field (0.05 Tesla) for 2 min.

Energy expenditure analysis was assessed by indirect calorimetry using the Oxymax CLAMS system (Comprehensive Lab Animal Monitoring System, Columbus, OH, USA) at the end of the study. The rats were food-deprived for 6 h for fasting recordings and were fed their corresponding diets for the next 18 h. The animals were placed in a polycarbonate plastic chamber with a constant air flow that was monitored by a mass-sensitive flow meter and analyzed by an O_2_ and CO_2_ sensor. Oxygen consumption and CO_2_ generation were continuously measured throughout the test. Respiratory exchange ratio (RER) was calculated as the volume of CO_2_ produced (VCO_2_ ml kg^−1^ h^−1^) divided by the volume of O_2_ consumed (VO_2_ ml kg^−1^ h^− 1^).

### Serum biochemical variables

Serum glucose was measured using a glucose analyzer (Model 2700, YSI Inc.). Serum triacylglycerols (TG) were measured with a commercial enzymatic colorimetric kit (DiaSys Diagnostic Systems International, Holzheim, Germany) in a Beckman DU 640 spectrophotometer. The serum FGF21 concentration was determined by ELISA (rat/mouse fibroblast growth factor-21 (FGF21) Elisa Kit, EMD Millipore).

### Quantitative real-time PCR

Total RNA was extracted from the liver, WAT, and BAT of rats as described by Chomczynski and Sacchi [29], and the RNA integrity, concentration, and purity were measured. The synthesis of cDNAs was performed with M-MLV reverse transcriptase and oligo-(dT)12–18 primer (Invitrogen). The concentrations of specific mRNAs were measured by real-time quantitative PCR using TaqMan Universal Master Mix (Applied Biosystems/Roche, Branchburg, NJ, USA) with an ABI Prism 7000 Sequence Detection System (Applied Biosystems, Foster City, CA). TaqMan fluorogenic probes and oligonucleotide primers were obtained from Applied Biosystems for the following genes: peroxisome proliferator-activated receptor coactivator 1-α (*Pgc-1α*; Rn00580241 m1), and T-box 1 (*Tbx1*; Rn01405403_m1) and uncoupling protein-1 (*Ucp1*; Rn00562126_m1). Hypoxanthine phosphoribosyltransferase (*Hprt*) (Rn01527840_m1) was used as the invariant control for BAT and WAT. The relative amounts of all mRNA samples were calculated using the comparative C_T_ method [30, 31]. The expression of PR domain containing 16 (*Prdm16*), tumor necrosis factor receptor superfamily, member 9 (*CD137/Tnfrs9*), cell death-inducing DNA fragmentation factor-α-like effector A (*Cidea*), *Fgf21*, and *Hprt* genes was determined by the SYBR green PCR kit (Roche) with the following pairs of primers: 5′-aggcccctgtctacattcct-3′ and 5′-tctcctgggatgacacctct-3′ for *Prdm16*, 5′-gacttcctcggctgtctcaa-3′ and 5′-ttctgtgtcacccagtgctc-3′ for *Cidea*, 5′-acaccgcagtccagaaagtc-3′ and 5′-caggcctcaggatcaaagtg-3′ for *CD137*, 5′-ctggtgaaaaggacctctcg-3′ and 5′-ggccacatcaacaggactct-3′ for *Hprt* as housekeeping gene. All primers were designed with at least one primer spanning an exon-exon boundary.

### Immunoblotting

Tissues were homogenized at 4 °C in ice-cold RIPA buffer containing phosphate-buffered saline (PBS), 1% IGEPAL, 0.5% sodium deoxycholate, 0.1% sodium dodecyl sulfate, 1 mmol/L sodium fluoride, 2 mmol/L sodium orthovanadate, and 1 tablet/10 mL of protease inhibitor mixture (Complete Mini, Roche Diagnostics). The extracts were stored at − 70 °C until further use. The protein concentration was determined with the Lowry method. Total protein from each rat tissue (20 μg each) was separated on a 7% SDS-polyacrylamide gel and transferred to a PVDF membrane (Hybond-P, Amersham) through electroblotting (Trans-Blot, Bio-Rad). The membranes were blocked for 1 h with 5% non-fat dry milk, washed 3 times for 5 min each with Tris- buffered saline containing 0.1% Tween (TBS-T), and incubated with primary antibody diluted in blocking solution overnight. Primary antibodies against the following proteins were used: TBX-1 (1:1000) (Santa Cruz Biotechnology), FGF21 (1:750), PGC-1α (1:250), and UCP1 (1:3000) (Abcam). The membranes were washed three times with TBS-T for 10 min and later incubated with horseradish peroxidase- conjugated secondary antibody (1:3500) for 1.5 h. Visualization was performed using a chemiluminescent detection reagent (Millipore, MA, USA). Digital images of the membranes were obtained by a ChemiDoc MP densitometer and processed by Image Lab software (Bio-Rad, Hercules, CA, USA). The results are reported relative to GAPDH (liver, BAT) and γ-tubulin (WAT and BAT). A value of 1 was arbitrarily assigned to the 20% room temperature group, which were used as a reference for the other conditions.

### Immunofluorescence analyses

Samples of BAT and subcutaneous WAT were dissected, immediately fixed with ice-cold 4% (*w*/*v*) paraformaldehyde in PBS, and embedded in paraffin, and sections of 4 μm were obtained. The sections were deparaffinized at 60 °C for 20 min, immersed in xylene, rehydrated through graded ethanol solutions, and finally in distilled water. The sections were washed with 1X PBS and blocked with 10% rabbit serum (Santa Cruz Biotechnology) for 30 min at room temperature. The sections were subsequently incubated with rabbit anti-UCP1 1:100 (Abcam) at room temperature for 1 h. After washing with 1X PBS, the sections were incubated with goat anti-rabbit FITC-conjugated secondary antibody (1:500; Santa Cruz Biotechnology) at room temperature for 1 h. The sections were washed again with 1X PBS, mounted with UltraCruz™ mounting medium (Santa Cruz Biotechnology), and viewed on a Leica DM750 microscope (Leica, Wetzlar, Germany).

### Statistical analysis

The values are expressed as the means ± SEM. Data were assessed using the Kolmogorov-Smirnov Z test to examine the distribution type; all results exhibited a normal distribution. Two-way ANOVA was used to determine the main effects of diet (% protein) and environmental temperature (room temperature 23 °C vs. cold exposure 4 °C) and their interaction. When a significant interaction effect was found, the differences between all groups were determined using Fisher’s protected least significant difference test. The data were analyzed by using GraphPad Prism (version 7.0 Graph Pad Software, Inc.). Analysis of covariance (ANCOVA) for energy expenditure and body weight in rats fed with different dietary protein concentrations was performed using SPSS for Mac (version 21). All analyses were performed at least 3 times to ensure reproducibility. The differences were considered statistically significant at *P* < 0.05. Mean values with different lowercase letters show statistical differences between each other (a> b> c> d> e).

## Results

### Body weight, food intake, and biochemical variables in rats fed with different amounts of dietary protein exposed at room and cold temperature

We first analyzed the effect of the amount of dietary protein on body weight gain and food intake in rats maintained at room temperature or exposed to a cold environment. The data showed that in those rats maintained at room temperature during the 3-day experimental period, there was no significant difference in body weight gain; however, rats fed 20% or 50% dietary protein tended to have a higher body weight than those fed 6%. Interestingly, rats exposed to a cold environment for 72 h had a significant reduction in body weight that did not differ among groups; however, those fed 50% dietary protein tended to lose less body weight (Table 2). The changes in the six experimental groups showed no significant difference in food intake expressed as gram per day or kilocalorie per day, since the diets were isocaloric. Nonetheless, the amount of protein consumed was according to the protein content of the diet. Notably, the amount of protein consumed for the corresponding groups under room or cold temperature conditions did not show a significant difference. Interestingly, despite the protein content in the diet or temperature differences, the rats maintained their percentage of fat and lean body mass. Serum blood glucose and triglycerides were significantly higher in rats exposed to a cold environment by approximately 9% and 69%, respectively, compared to those maintained at room temperature, without a significant difference with regard to the protein content of the diet.

**Table 2:** Food intake and serum hormonal and biochemical variables in rats fed different protein/carbohydrate ratios and exposed for 72 h to a cold environment

	Room temperature (23 °C)		Cold temperature (4 °C)				Protein (%)	Temperature (°C)	Interaction DP X T
	Protein content in the diet (%)								
	6	20	50	6	20	50			
Δ Body weight (g)	10.2 ± 1.5	17.3 ± 3.9	13.5 ± 6.7	− 26 ± 9.6	− 22.8 ± 4.8	− 16.8 ± 6.6	NS	< 0.0001	NS
Food intake (g/d)	32.9 ± 1.5	29.0 ± 3.2	30.6 ± 2.0	34.1 ± 1.9	34.2 ± 1.8	32.6 ± 1.0	NS	NS	NS
Food intake (kcal)	115.2 ± 5.3	101.5 ± 11.2	107.1 ± 7	119.4 ± 6.7	119.7 ± 6.3	114.1 ± 3.5	NS	NS	NS
Protein intake (g/d)	2.0 ± 0.1	5.8 ± 0.6	15.3 ± 1	2.1 ± 0.1	6.8 ± 0.4	16.3 ± 0.5	< 0.0001	NS	NS
Protein intake (kcal/d)	8.0 ± 0.4	23.2 ± 2.6	61.2 ± 4	8.2 ± 0.5	27.3 ± 1.4	65.2 ± 2	< 0.0001	NS	NS
Glucose (mM)	7.4 ± 0.12	8.1 ± 0.22	8.0 ± 0.14	8.6 ± 0.19	8.6 ± 0.3	8.5 ± 0.2	NS	< 0.0001	NS
Triacylglycerols (mM)	2.2 ± 0.14	2.5 ± 0.31	1.81 ± 0.11	3.65 ± 0.23	3.95 ± 0.3	3.44 ± 0.92	NS	< 0.0001	NS
Fat body mass (%)	12.3 ± 0.7	10.7 ± 1.1	11.8 ± 0.9	11.1 ± 2.5	10.2 ± 1.0	10.4 ± 2.2	NS	NS	NS

Values are the mean ± SEM, *n* = 5 rats per group. Protein-temperature interaction was evaluated by two-way ANOVA. Significant differences for % dietary protein, temperature, and the interaction between them (DP X T) are reported as significant (*p* < 0.05) or not significant (NS)

### Expression of WAT browning markers in rats fed with different amounts of dietary protein exposed at room and cold temperature

**Fig. 2:**
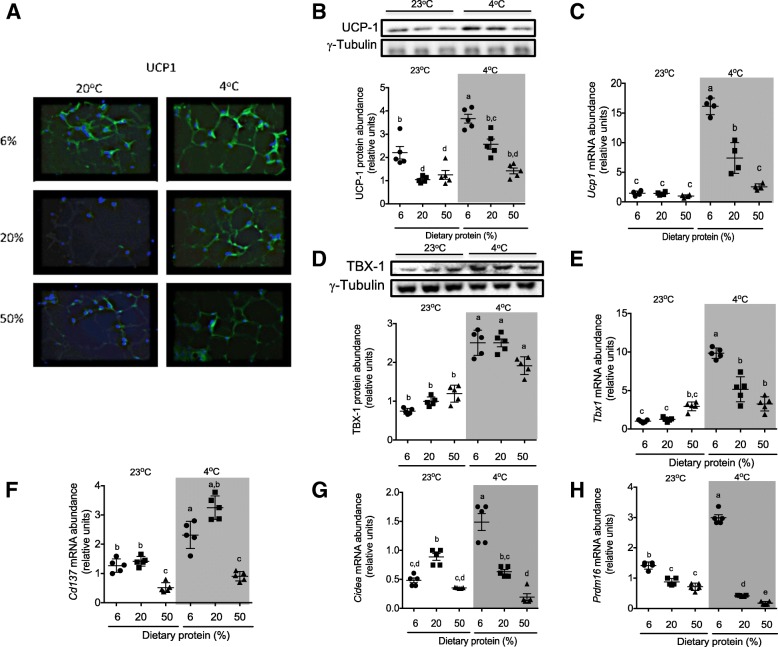
Expression of UCP1 and browning markers in WAT. **a** UCP1 immunofluorescence. Protein abundance and densitometry analysis normalized to γ-tubulin of **b** UCP1 and **d** TBX1. Relative mRNA abundance of **c** *Ucp1*, **e** *Tbx1*, **f** *Cd137*, **g** *Cidea*, and **h** *Prdm16* all normalized to hypoxanthine phosphoribosyltransferase (HPRT) of WAT. Subcutaneous adipose tissue from inguinal area was obtained from rats fed low (6%), adequate (20%), and high (50%) dietary protein and maintained at room temperature (23 °C) or exposed to a cold environment (4 °C) for 72 h. All mRNA and protein abundance data are expressed relative to rats fed an adequate protein diet at room temperature. The results are presented as the means ± SEM, *n* = 5 rats per group. The differences were considered statistically significant at *P* < 0.05. Mean values with different lowercase letters show statistical differences between each other (a > b> c > d)

Next, we assessed the effect of the consumption of different amounts of dietary protein on the WAT browning. As expected, rats exposed to a cold environment showed a significantly increased expression of UCP1 and all of the browning markers, particularly TBX1 (Fig. 2d, e). Interestingly, these data clearly showed that the consumption of a low-protein diet under a cold environment greatly increased UCP1 abundance, whereas at room temperature, this increase was less evident as observed in the immunofluorescence staining (Fig. 2a), Western blot analysis (Fig. 2b), and real-time PCR (Fig. 2c). However, this response was highly repressed after the consumption of a high-protein diet, despite the cold temperature. Furthermore, the increase in the expression of *Ucp1* was accompanied by the significantly higher expression of other browning markers, such as *CD137* (Fig. 2f), *Cidea* (Fig. 2g), and *Prdm16* (Fig. 2h).

### Expression of thermogenic genes in BAT of rats fed with different amounts of dietary protein exposed at room and cold temperature

**Fig. 3:**
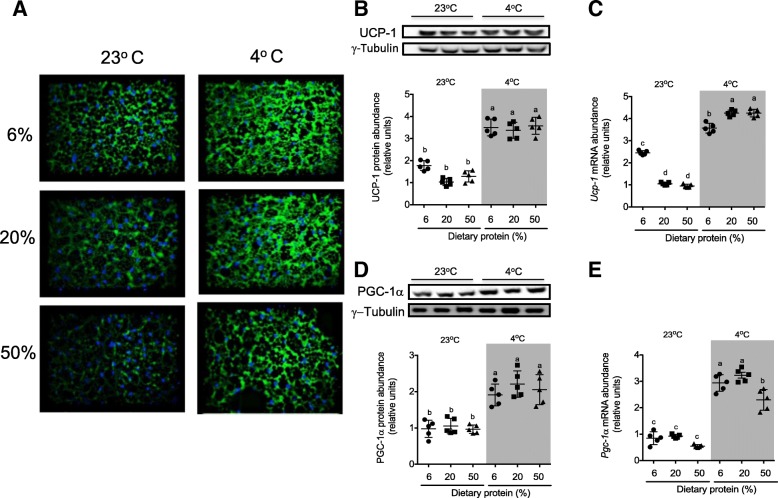
mRNA abundance and protein expression of genes associated with thermogenesis in BAT. **a** UCP1 immunofluorescence. Representative blots and densitometry analysis normalized to γ-tubulin of **b** UCP1 and **d** PGC-1α. Relative mRNA abundance of **c** *Ucp1* and **e** *Pgc-1*훼*.* Brown adipose tissue was obtained from rats fed low (6%), adequate (20%), and high (50%) dietary protein and maintained at room temperature (23 °C) or exposed to a cold environment (4 °C) for 72 h. All mRNA and protein abundance data are expressed relative to rats fed an adequate protein diet at room temperature. The results are presented as the means ± SEM, *n* = 5 rats per group. The differences were considered statistically significant at *P* < 0.05. Mean values with different lowercase letters show statistical differences between each other (a > b > c> d)

Next, we explored whether the amount of dietary protein modulates *Ucp1* and *Pgc1α* mRNA abundance and protein content in BAT. As expected, cold exposure increased the expression of both thermogenic indicators independently of the protein content in the diet (Fig. 3a–e). As observed in WAT, UCP1 protein expression was higher in the BAT of rats fed a low- protein diet and maintained at room temperature, compared to those fed an adequate or high-protein diet (Fig. 3a–c). However, PGC1α was not modified under these conditions (Fig. 3d, e).

### Circulating concentrations of FGF21 in rats fed with different amounts of dietary protein exposed at room and cold temperature

**Fig. 4:**
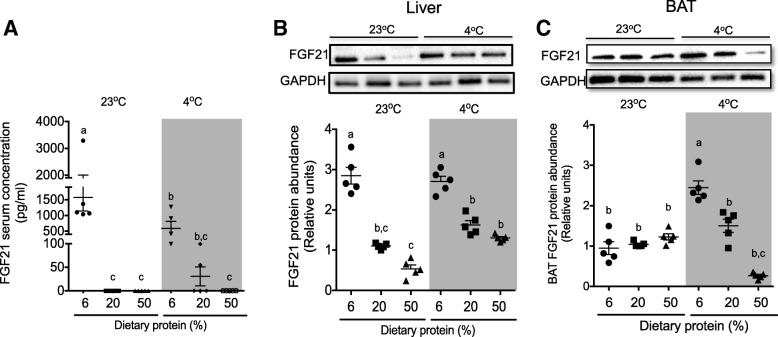
FGF21 serum concentration and expression in liver and BAT. **a** FGF21 serum concentration, **b** hepatic FGF21 relative protein abundance, representative blot and densitometry analysis normalized to GAPDH, and **c** BAT FGF21 relative protein abundance, representative blot and densitometry analysis normalized to GAPDH. Serum, liver, and brown adipose tissue were obtained from rats fed low (6%), adequate (20%), and high (50%) dietary protein and maintained at room temperature (23 °C) or exposed to a cold environment (4 °C) for 72 h. Protein abundance data are expressed relative to rats fed an adequate protein diet at room temperature. The results are presented as the means ± SEM, *n* = 5 rats per group. The differences were considered statistically significant at *P* < 0.05. Mean values with different lowercase letters show statistical differences between each other (a > b > c > d> e)

Cold exposure and a low-protein diet increase circulating levels of FGF21; however, it is not known whether both factors have a synergistic effect on the FGF21 serum concentration [32]. Therefore, we measured the circulating concentration of FGF21 in the serum of rats fed low-, adequate, or high-protein diets at room or cold temperature. Rats fed a low-protein diet showed higher serum FGF21 concentration. Rats exposed to a cold environment also presented a higher FGF21 concentration when fed a low-protein diet; however, rats at cold temperature had lower circulating levels than those at room temperature (*P* < 0.05). The increase of dietary protein above 20% strongly suppressed circulating FGF21. Intriguingly, this response was almost abolished when rats were fed a high- protein diet independently of environmental temperature (Fig. 4a).

### Hepatic expression of FGF21 in rats fed with different amounts of dietary protein exposed at room and cold temperature

To understand how the changes in the magnitude of circulating FGF21 concentration in rats fed different amounts of dietary protein at room or cold temperature were associated with changes in FGF21 expression in the liver, we studied hepatic FGF21 protein abundance. Hepatic FGF21 protein abundance increased 1.8-fold in rats fed a low- protein diet compared to those fed adequate or high- protein diets at room temperature. Rats fed a low- protein diet and exposed to a cold environment showed a similar FGF21 protein abundance compared to those maintained at room temperature, but its abundance was reduced in the liver of rats fed a high-protein diet.

### Expression of FGF21 in BAT of rats fed with different amounts of dietary protein exposed at room and cold temperature

In addition, there is evidence that a second source of FGF21 is the BAT. It is clearly observed from Fig. 4c that FGF21 protein abundance did not increase when rats were fed a low-protein diet at room temperature. Cold exposure increased BAT FGF21 in rats fed 6% and 20% dietary protein compared to those maintained at room temperature. However, FGF21 protein abundance was highly repressed when rats consumed 50% of dietary protein.

### Energy expenditure and respiratory exchange ratio in rats fed with different amounts of dietary protein exposed at room and cold temperature

**Fig. 5:**
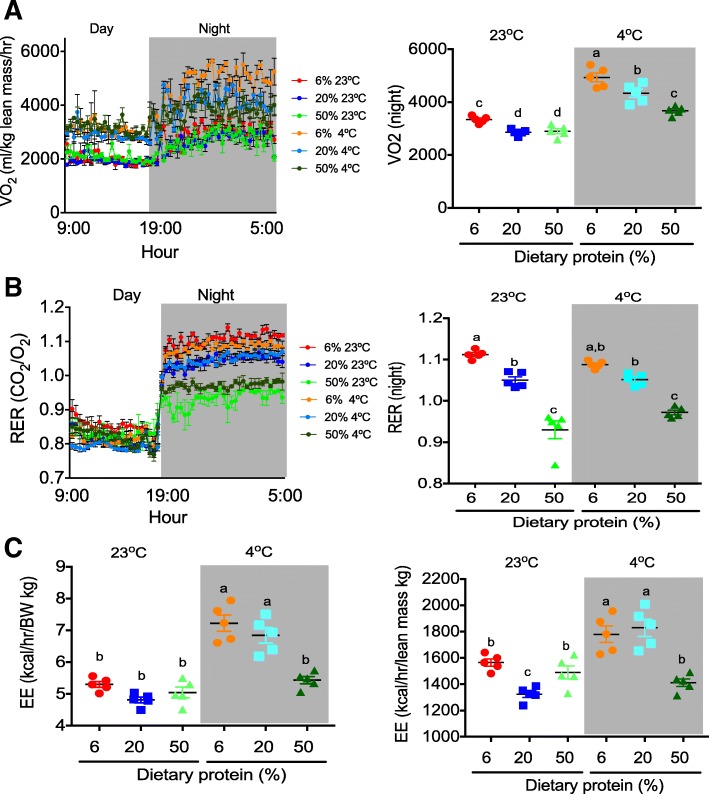
Whole-body energy expenditure. **a** Oxygen consumption, **b** respiratory exchange ratio (RER), and **c** energy expenditure of rats fed low (6%), adequate (20%), and high (50%) dietary protein and maintained at room temperature (23 °C) or exposed to a cold environment (4 °C) for 72 h (*n* = 5 rats per group). Energy expenditure (**c** left) was corrected for body weight. Oxygen consumption and energy expenditure (**c** right) were normalized to lean mass. The differences were considered statistically significant at *P* < 0.05. Mean values with different lowercase letters show statistical differences between each other (a > b > c > d > e)

To evaluate the physiological significance of increased markers of WAT browning and BAT thermogenesis, RER and oxygen consumption were determined by indirect calorimetry. Indirect calorimetry showed that those animals fed a low-protein diet at room temperature had a significant increase in oxygen consumption and energy expenditure compared to those fed higher amounts of dietary protein, suggesting that FGF21 may induce a mechanism to increase energy expenditure by the browning of adipose tissue, as previously reported [33]. When rats were exposed to cold temperature, those fed a low-protein diet also had higher oxygen consumption (Fig. 5a) and energy expenditure even when normalized to lean body mass (Fig. 5c), despite lower FGF21 circulating levels (Fig. 4a). Interestingly, regardless of the environmental temperature, rats fed a low-protein diet showed a RER value above 1 (Fig. 5b). As expected, rats fed adequate protein diets showed a RER of approximately 1.0, indicative of glucose utilization as an energy substrate, whereas rats fed a high- protein diet had a 0.9 RER, showing that an excess of amino acids is used as energy substrates (Fig. 5b).

## Discussion

Due to the worldwide epidemic of obesity, several mechanisms of energy expenditure regulation have been extensively studied [34, 35]. Diet has been investigated as a key element in the regulation of the energy balance [36–39]. Several studies have demonstrated that dietary protein consumption can affect energy expenditure; however, the results have been contradictory, due to a wide range of experimental designs including length of exposure to the diet, type and amount of protein, species, etc. [24, 40–43]. Studies in humans show that total energy expenditure is significantly lower in subjects fed with the low protein compared to those fed adequate or high-protein diet [40]. They suggest that changes in body composition in lean body mass and fat storage due to the consumption of different amounts of dietary protein can alter energy expenditure. In our study, we did not observe significant changes in body composition in rats fed different concentrations of dietary protein in part due to the short period of exposure to the experimental conditions (Table 2). However, another study did not show an effect on postprandial resting energy expenditure in subjects fed different amounts of dietary protein [42]. A study in rats shows that switching diets from an adequate protein diet (14%) to a high- protein diet (55%) for 1, 3, 6, or 14 days did not change total or resting energy expenditure, but there was only an increase in respiratory quotient after diet switching [43]. This indicates that the time of adaptation to changes in the diet is very important and thus affects energy expenditure observations, explaining in part controversial results in animals and humans. The present study showed that the consumption of a low-protein diet at room temperature increased energy expenditure. Recent evidence has linked FGF21 with energy expenditure by its ability to activate WAT browning and thermogenesis [32]. Our results showed that the increase in circulating FGF21 in rats fed a low-protein diet was associated with an increase in *Ucp1* mRNA and protein expression in WAT. These results are in accordance with recent evidence that has demonstrated that the consumption of a low-protein diet increases energy expenditure by the effect of FGF21 on UCP1 activity, since deletion of UCP1 blunts the low-protein diet effect on energy expenditure [24, 44]. Laeger et al. [13, 45] demonstrated that LP diets increased not only energy expenditure in mice, but also WAT *Ucp 1* and *Cidea* expression, and by histological assessment, they found morphological changes such as multilocular adipocytes, data that is consistent with browning. Furthermore, these observations were not observed in *Fgf21* KO mice. They suggest that the amino acid sensor ATF4/GCN2 may be the link towards the increase in FGF21 during amino acid restriction, but only in a short-term response. Notably, rats exposed to a cold environment fed a low-protein diet showed lower circulating FGF21 than those at room temperature, but had higher expression of WAT browning markers suggesting that the increase in energy expenditure in rats fed a low-protein diet and maintained at cold temperature involves other mechanisms besides FGF21. Recently, Ameka et al. [46] showed that hepatic FGF21 is induced by acute but not chronic cold exposure, and it signals the CNS to regulate the sympathetic nervous system in BAT and control body temperature. They proved by using liver-specific or WAT- specific FGF21 KO mice that only liver FGF21 is released to circulation after cold exposure. Also, they demonstrated that central FGF21 signaling is necessary for thermoregulation in BAT during cold exposure.

Also, there is evidence that regulation of thermogenesis may include another FGF21-independent mechanism as suggested by Keipert et al. [47], who showed that mice deficient in FGF21 present WAT browning during cold exposure. Interestingly, it has been demonstrated that the consumption of a high-protein diet reduces the β-adrenergic response in WAT [48]. Several lines of research may explain this finding: (a) It is known that the activation of α-1-adrenergic receptor downregulates hepatic FGF1 synthesis and circulating levels [49]. (b) It was shown that cold exposure increases α-1-adrenergic receptor in BAT [50]; however, it is not known if this also occurs in the liver. This may explain the FGF21 differences between room temperature and cold exposure. However, this mechanism should be explored in detail in future studies.

Furthermore, the present data clearly showed that consumption of an adequate or a high-protein diet at room temperature decreased the expression of FGF21. These results are consistent with those of a previous study that has demonstrated that a high-protein diet decreases the expression and circulating levels of FGF21 [51]. Protein restriction activates the GCN2/pEIF2α/ATF4 pathway involved in the upregulation of FGF21 gene expression [13], and the high availability of amino acids from a high-protein diet may partially suppress this signaling pathway.

Our results are in agreement with previous studies that have shown that FGF21 is only released when dietary protein is low, and there is no difference if it is accompanied with high-fat or high-carbohydrate diet [52, 53], effect that was not observed when mice were fed with adequate or high-protein diets in combination with different concentrations of fat or carbohydrate in the diet. In fact, these studies showed that restricting energy intake without protein restriction fails to increase FGF21 [13].

Contrary to the effect of a low-protein diet, we observed a remarkable inhibitory effect of a high-protein diet in cold-stimulated WAT browning. This result suggests that in WAT, cold exposure and amino acid availability exert antagonistic effects on browning. In fact, a recent study shows that WAT browning is negatively regulated by the FLCN-mTOR-TFE3-PGC-1β pathway that is modulated by an increase in amino acid availability [54]. However, during cold exposure, β-adrenergic activation of mTOR through PKA leads to S6K phosphorylation and WAT browning [55]. Thus, these results indicate that an increase in dietary protein content and cold exposure exert opposite effects on energy balance through the modulation of WAT browning.

Similarly, dietary protein content modulated UCP1 abundance in BAT at room temperature. However, during cold exposure, UCP1 abundance was exclusively regulated by environmental temperature and did not respond to dietary protein intervention. In addition, there is evidence that BAT synthesizes and releases FGF21 [16, 19]. In fact, we demonstrated that FGF21 is expressed in BAT. Nonetheless, although FGF21 protein abundance is upregulated when rats are fed a low or adequate protein diet during cold exposure, this increase did not have a significant influence on FGF21 circulating levels. Thus, the role of FGF21 in BAT is mostly autocrine, as previously reported [16, 19, 56].

The present evidence suggests that low-protein diets can stimulate energy expenditure via FGF21 and WAT browning and could be used as a dietary strategy for obesity treatment. Recent evidence has demonstrated that this biological effect likely occurs in obese rat models [45, 57–59]; however, the long-term effects of this dietary strategy should be carefully evaluated, since long-term low-protein diets can stimulate the appearance of fatty liver [44], and moderate restriction can modify the gut microbiota, which in turn can affect energy harvesting capacity [60, 61], as well as the WAT browning process.

## Conclusions

In summary, the present results indicate that in addition to the environmental temperature, WAT browning is nutritionally activated by low dietary protein, increasing whole-body energy expenditure, whereas a high-protein diet can repress this effect. More studies are needed to further understand the mechanism by which low dietary protein mediates the increase in WAT browning and use this strategy in obese human subjects to increase energy expenditure and reduce body weight and fat mass to ameliorate the metabolic abnormalities of obesity.

## Abbreviations

BAT: Brown adipose tissue
CIDEA: Cell death-inducing DNA fragmentation factor-α-like effector A
FGF21: Fibroblast growth factor 21
HPRT: Hypoxanthine phosphoribosyl transferase
PGC-1α: Peroxisome proliferator-activated receptor coactivator-1α
PPAR: Peroxisome proliferator-activated receptor
PRDM16: PR domain containing 16
TBX-1: T-Box-1
UCP1: Uncoupling protein-1
WAT: White adipose tissue
